# A Qualitative Application of Temporal Self-Regulation Theory to Understand Adherence to Simple and Complex Medication Regimens

**DOI:** 10.3390/healthcare8040487

**Published:** 2020-11-16

**Authors:** Caitlin Liddelow, Barbara Mullan, Mark Boyes, Hannah McBride

**Affiliations:** Health Psychology and Behavioural Medicine Research Group, School of Psychology, Curtin University, Bentley, Western Australia 6021, Australia; caitlin.liddelow@postgrad.curtin.edu.au (C.L.); mark.boyes@curtin.edu.au (M.B.); hannah.mcbride@postgrad.curtin.edu.au (H.M.)

**Keywords:** temporal self-regulation theory, medication adherence, complexity, routines, cues, planning

## Abstract

Medication adherence is a global health concern, and variables of temporal self-regulation theory (TST) have been shown to be important in improving adherence. This qualitative study aims to explore how TST can help explain medication adherence in people’s daily lives, and whether there are differences in the adherence to simple and complex medication regimens. Twenty-nine participants from Australia engaged in semi-structured interviews based on TST (intention, behavioural prepotency, self-regulation), and other variables important to adherence. Interviews were analysed using thematic analysis. Six themes were identified (Routines, External Supports, Cost, Sense of Agency, Adverse Outcomes, and Weighing Up Pros and Cons), with partial support for TST (specifically intention, past behaviour, cues and planning). Four themes not related to TST were also identified. Individuals with more complex medication regimens spoke of the importance of routines, planning, and knowledge-seeking, whereas those with simpler regimens spoke of the importance of visual cues. TST may be useful for identifying some variables important in medication adherence, however, additional factors were also identified. For simple regimens, future research should focus on the manipulation of visual cues. For complex regimens, health professionals should consider supporting the use of medication management apps to assist in planning and ensuring a consistent routine.

## 1. Introduction

In 2003, the World Health Organization (WHO) declared medication non-adherence as a worldwide issue of striking magnitude, which should be a priority for policymakers and health care providers [1]. Despite this, medication adherence remains a global health concern [2]. It is estimated that in developed countries, approximately 50% of adults with a chronic illness do not adhere to their medication regimens [3], with this number expected to be even lower for preventative medications such as the oral contraceptive pill, which do not provide instant symptom relief [4]. Poor medication adherence increases the likelihood of experiencing adverse medical events, worsening of condition symptoms, increased comorbidity, higher health care costs, and in some instances, higher risk of mortality [5]. Similarly, medication non-adherence creates an economic burden, costing approximately USD 100–300 billion in the United States alone [6]. With an increase in the number of co-morbid chronic diseases in adults, particularly young adults [7], including diabetes, hypertension, mental health disorders, Crohn’s disease and arthritis [8,9,10] it is important set up healthy and effective adherence patterns in young adulthood. The transition to adulthood is a difficult time for many, and a time where many begin to take control over their own health, however this population tends to be underrepresented in the adherence literature [11,12], with most research recruiting older adults.

There is a large body of scientific literature investigating medication adherence, or lack of adherence, using quantitative research methods. Many of these existing quantitative studies have employed the use of health psychology theories to explain non-adherence [13], and to guide intervention creation in attempts to improve the behaviour [14]. In attempting to understand medication adherence, these quantitative studies have identified the role of important mechanisms such as self-efficacy, perceived barriers, perceived susceptibility, necessity beliefs, and concerns about medication [13]. Many of the theories have also been extended in attempts to negate weak relationships between variables (i.e., intention and behaviour in the theory of planned behaviour) [15] or to explore the influence of non-psychosocial variables in medication adherence (i.e., side effects) [16]. However, whilst these variables may be important for predicting adherence to various types of medications, it is not known which variables are more or less influential in predicting adherence to regimens of varying complexities. Complex medication regimens are commonly defined as taking at least five medications at one time [17].

Temporal self-regulation theory (TST) [18] is a newer theory of behaviour change that incorporates dual processes (e.g., rational and automatic processes) in the prediction of behaviour. The theory extends from the primary premise of the theory of planned behaviour which suggests that intention is the most proximal predictor of behaviour [19] by incorporating additional processes, i.e., self-regulatory capacity (rational processes) and behavioural prepotency (automatic processes). Rational or conscious processes are those under volitional control, such as intention and some self-regulatory processes like planning, goal-setting and self-efficacy [20]. Automatic or unconscious processes (i.e., habit) operate without conscious awareness and tend to be executed without thought [21]. The theory proposes that intention, behavioural prepotency and self-regulatory capacity all directly predict behaviour. In addition, both behavioural prepotency and self-regulatory capacity are proposed to moderate the intention–behaviour relationship [18].

Although temporal self-regulation theory has been quantitatively applied to various health behaviours [22,23,24] it has only been applied to medication adherence in one study [16]. In this study, the theory predicted approximately 50% of the variance in adherence to a range of medication types and regimen complexities, with both rational (intention, planning and self-control) and automatic (habit and cues) processes being important. However, this study did not individually investigate the predictors of adherence to simple or complex regimens. Certain variables in temporal self-regulation theory may be more important in simple regimens, rather than complex regimens, and vice versa. For instance, habit may be more predictive of adherence to a simple regimen, as taking a single medication at the same time every day is likely to become more habitual, and the repetition of a single action within the same context is an optimum environment for habits to form [21]. Similarly, more complex medication regimens have been associated with decreased adherence [25]. Thus, having a greater understanding of the modifiable predictors that may be associated with increased adherence to these complex regimens is important, not only for researchers tailoring interventions, but also general practitioners and pharmacists. One way to go about this is through the use of qualitative research methods, as quantitative explorations of behaviour change theories can only tell us what variables may be important in a behaviour but lack the “why”. Qualitative research also allows for the identification of additional variables that may not be accounted for in the theory, and may be capable at predicting additional variance when tested quantitatively. Furthermore, qualitative research is important in medication adherence research as it allows both researchers and clinicians to further understand medication adherence, or non-adherence, from the patient’s point of view, rather than just their responses to self-report measures, which may be biased [26].

By focusing research on the modifiable psychological variables associated with adherence to different medication regimen complexities, interventions aimed at increasing adherence to various regimen complexities can be tailored. In addition, by having a further understanding of the influence of the variables that may not be modifiable or accounted for in behaviour change theories (e.g., side-effects), findings regarding these can be communicated with clinicians who can then provide their patients with ways of navigating and living with such effects, or be considered when designing interventions to improve adherence [27].

### The Current Study

To guide this qualitative research study, the overarching research questions “what variables from temporal self-regulation theory are important in adhering to different medication regimens, specifically simple and more complex regimens?” and “are there any additional variables, not included in the theory, that are important in effectively adhering to these different regimens?” were explored. The overarching aim was to explore how temporal self-regulation can help explain medication adherence in people’s daily lives, and whether the theory explains different patterns of behaviour in the adherence to simple and complex medication regimens. An inductive and deductive qualitative approach was used to ensure the possibility of identifying other additional variables that are important in adherence, and that are not accounted for in the theory. As temporal self-regulation theory only accounts for a moderate amount of variance, the identification of additional influences is just as important.

## 2. Methods

### 2.1. Participants

Participants were recruited through the University participant pool for undergraduate students and received course credit to thank them for their time. All participants were required to be over the age of 16 years and be taking regular prescription medication for an ongoing health concern.

### 2.2. Interview Schedule

A semi-structured interview schedule (see Supplementary Materials) was developed for this study. The interview questions were based on the temporal self-regulation theory constructs—intention, habit, past behaviour and self-regulatory capacity, and also included questions related to their experience of side-effects, current regimen and support. All questions were open-ended and had several prompts associated with them to continue the discussion.

### 2.3. Procedure

Ethical approval for this study was obtained from the University’s Human Research Ethics Committee (HRE2017-0173). Participants provided consent by checking a box in Qualtrics, completed a demographic questionnaire, and were invited to attend a face-to-face interview. Interviews lasted for approximately 30 min and were conducted until the interviewees no longer presented or discussed new information. All interviews were transcribed verbatim by an independent transcriber not associated with the study. After transcription, all identifying information was removed.

### 2.4. Data Analysis

Thematic analysis [28] taking both a deductive and inductive approach, was applied to analyse the data through coding and the identifying of prominent patterns or themes. All analysis was conducted by hand by two researchers. Each transcript was read through by both researchers to aid familiarisation. One researcher read each transcript line-by-line, interpreting analytic codes. The second researcher then further refined these codes into latent codes (preliminary themes). Both researchers met many times to further refine the latent codes into themes and subthemes to capture the underlying ideas present in the data. This process was conducted until no further themes could be identified [29,30]. The identified themes were checked by two additional researchers for review and feedback to strengthen the trustworthiness and dependability of the findings.

### 2.5. Quality Procedures

To ensure quality and rigor, each participant was sent a transcribed version of their interview for member checking [31], to ensure accuracy of the transcribed data to the participants’ experiences and views [32]. In addition, triangulation was used to reduce potential researcher subjectivity and bias in the interpretations and analysis of the data, and subsequently increases the trustworthiness of the study and findings [32]. Any discrepancies or disagreements in analysis were verbally discussed and resolved. Throughout the analysis process, the researchers kept notes related to any broader concepts or ideas that seemed to underly the themes.

## 3. Findings

### 3.1. Participants

A total of 29 participants were interviewed, with the average number of participants in similar research being 33 participants (range = 7–98) [33]. Of these, 4 identified as male and 25 identified as female with ages ranging from 18 to 52 years (*M* = 20.16 years). Participants reported having between one and five medications in their regimen. Regimens included a range of medication forms (i.e., tablets, injections, and inhalations), dosages (i.e., once daily, when needed, every other day) and a variety of health issues (e.g., acne/skin problems, depression, polycystic ovary syndrome/endometriosis, asthma, diabetes, obsessive-compulsive disorder, and anxiety).

### 3.2. Themes

#### 3.2.1. Routines

The theme “Routines” describes participants’ process of purposefully implementing plans to assist in them maintaining their medication regimens in a consistent and routinised way. These routines appeared to be characterised by participants consciously putting plans into place, and the use of cues like setting an alarm or putting their medication in an easily visible location. Within this sample, this deliberate routinisation appeared to be more salient for more complex regimens (i.e., those managing a regimen of three to five different medications). For instance, one participant with a complex regimen commented:

*Yeah just because I take it in the morning, so then like it’s easy just to take two at the same time.*

(Participant 22, p. 4)

Here, the participant appeared to highlight that a simple routine, taking all medications at one time, was convenient and assisted in effective adherence. Furthermore, participants noted that significant changes to their routine appeared to impact their adherence in that they were more inclined to miss their medication after a routine change. For example, one participant, whose regimen changed from taking all medications in the morning to taking one of them at night, said:

*So when I added my, when I used to take the (medication) in the morning, that was the easiest, just to remember to take them both together. But now the other one is at night. So yes more, a bit more inclined to forget at night-time.*

(Participant 26, p. 4)

##### Planning

This subtheme describes participants’ efforts to purposefully organise certain aspects of their medication routines, such as managing their prescriptions and finding the time to go to the pharmacy/chemist. Planning seemed especially important to participants taking multiple medications. This was outlined where one participant, with a regimen of five different medications, commented:

…*having to find the time to go into the chemist, remembering to refill your scripts … I usually try to have everything ready *(prescriptions refilled)* when I have, you know, maybe four days left*.

(Participant 23, p. 5)

Some participants also mentioned planning to take medication ahead of any events that might clash with their usual routine. For instance, one noted:

…*when I used to go out and be a party animal. I’d generally take it before I go*…

(Participant 5, p. 7)

##### Cues

In this subtheme, many participants, irrespective of the complexity of their regimen, mentioned cues that prompted them to take their medication. Such cues included visual cues (e.g., medication placement), sensory cues (e.g., taste), internal cues (e.g., noticing changes in one’s own emotional or cognitive state), and contextual cues (e.g., other medications taken or behaviours carried out simultaneously). For example, one participant spoke about incorporating a glass of water (a visual cue) into their morning routine:

…*and then the glass of water is there. It’s like, just take my tablets with that. So it’s sort of, the water is there to remind me I haven’t had them yet*

(Participant 14, p. 4)

Similarly, another participant commented that the flavour of one of their medications served as a prompt:

… *it also has a flavour, so it’s kind of like a tutti-frutti flavour. Whereas before I used to go to sleep with the taste of mint in my mouth from brushing my teeth. Now I have this sweet flavour in my mouth. So, if I lie down, I’m like ‘okay, time to go to sleep’, I’m like ‘oh, my mouth tastes normal’, go to the kitchen, and get it (*medication*)*

(Participant 22, p. 6)

Additionally, those whose regimens consisted of taking more than one medication noted that taking one medication would prompt taking the other/s and perhaps even improve their adherence. This is observed where one participant commented:

…*it probably helped me like not forget it as much. Because when I was just taking (*medication*), you know it was probably the one medication I didn’t have to have at a specific time. So I was just a little bit more ‘meh’ about it. So I guess now I don’t forget it as often.*

(Participant 17, p. 6)

##### Lifestyle Factors

In this subtheme, participants described various lifestyle factors as either barriers (e.g., work or social events) or facilitators (e.g., a consistent schedule) to adhering to their medication regimen. For example, one participant said:

…*sometimes I’m like ‘oh, I’ll stay at yours tonight” when I’m already out, and it (*medication*) has to be refrigerated because it like melts. So like, it dissolves in your mouth, but it also dissolves if there’s too much humidity or if it gets too hot. So I’m like, it’s a bit of a trek.*

(Participant 22, p. 7)

It appeared that this participant perceived their social life to have an adverse impact on their ability to adhere to taking their night-time medication. Conversely, another participant stated:

*I think in the morning I do have a routine, especially when uni is on or whenever I have study to do. I would wake up you know get breakfast and go take the pill*.

(Participant 26, p. 4)

This participant appeared to indicate that their consistent everyday schedule (i.e., lifestyle), may be conducive to their medication adherence.

#### 3.2.2. External Supports

The theme “External Supports” encompasses the influence of different support systems in participants’ adherence to their medication regimens, regardless of the complexity. Participants described receiving support in various areas of their life, such as from health professionals, family, and friends. One participant commented on the variety of support they receive and the interaction between them:

*My family are very different; they’ve been very supportive with my diabetes. They don’t really understand the academia side of things but my friends understand the academia and not so much the diabetes side and then my partner is a bit of both. So it’s kind of like I just get different support from different networks and it kind of sort of marries out.*

(Participant 27, p. 9)

It seems that where participants perceive support from those around them, they may feel less isolated or alone in their experiences of trying medication and creating a regimen. 

##### Professional Support

This subtheme describes participants’ experiences of receiving advice and knowledge from health professionals with regards to their medication regimen. For example, one participant stated:

…*and the bulk billing doctor I saw, she was really helpful. Told me all these things I didn’t even know*.

(Participant 19, p. 5)

Participants also spoke about being comfortable seeking further information from a trusted health professional. For instance, one participant stated:

*So yeah I’m pretty open to talking to my doctor about anything you know that could be wrong. Like side-effects and that kind of stuff*

(Participant 2, p. 6)

##### Social Support

In this subtheme, participants expressed feeling positive about having the support of significant others, such as family, romantic partners, and friends. As an example of this, one participant said:

*So classic mum drills it in. She’s like ‘You’ve got to take it, you know you’ll get sick and this and that will happen’. So she’s definitely supportive of it*…

(Participant 20, p. 4)

It appeared that this kind of support may aid in medication adherence for some people. Several participants also suggested that family and friends’ past experiences of taking medication may have been a salient element in their expressions of support, thus exhibiting more understanding and empathy. This was noted by one participant who stated:

*Mum has always encouraged it because she has been iron deficient since she was a little kid and yeah she knows the facts of it. And she’s always like “Go and get your blood tested for your iron levels” but she’s always just been conscious of it.*

(Participant 6, p. 4)

Some participants also expressed actively seeking support from loved ones to assist in adhering to their medication, and perhaps how this support is perceived as a positive factor in their adherence. For instance, one participant explained:

…*I guess he (partner) just didn’t really know (about taking the antidepressant)…Well he knew but like he didn’t think about it or anything but until I said a couple of months ago about, ‘Can you help me remember because I’m forgetting? And it’s important that I take it’*.

(Participant 18, p. 8)

##### Stigma

As a subtheme to “External Supports”, in contrast to receiving social support, some participants spoke about their experiences of stigma and stereotyping of their health concerns and medications by those around them, and thus this stigmatisation felt like a lack of support. For instance, one participant expressed:

*Mum wasn’t too sure of the pill *(contraceptive pill)*. Yeah, she obviously thought about the other connotations of me taking the pill and then got a bit upset*…

(Participant 26, p. 6)

Participants also expressed instances where they felt frustration or annoyance when experiencing stigma from loved ones, such that some of these instances invalidated their genuine emotional experiences, for example:

…*my parents would be like annoying if any small issue arises, they’d be like ‘have you taken it?’. As if like, ‘you’re acting irrational, have you taken it?’. Which like obviously wasn’t…like I had (taken the medication) so that was kind of like annoying*

(Participant 18, p. 5)

However, this frustration does not appear to have influenced the participant’s adherence to their medication. In addition, self-stigma also appeared evident in some interviews, such that it seemed to influence participants motivations to both start a medication and continue taking it. For example, a participant expressed:

*I was like I don’t need to take that…And I was like no, people who take this are really sick and that’s not me. And he was like okay sure, here’s a script, think about it.*

(Participant 7, p. 1)

#### 3.2.3. Cost

There did not appear to be any differences in the experience of participants of varying regimen complexities, despite some participants having to purchase a larger number of prescribed medications. Some participants spoke positively about their medication being cheap or subsidised, and how they felt fortunate. For example, one participant said:

*So this way *(purchasing the generic brand medication)* I can sort of like go and like it doesn’t make me want to be like ‘Oh, I’ll have to go pay for it’…At least this way it’s like easier to stick to and stuff. Like it’s cheaper, it’s easier all that kind of stuff yeah.*

(Participant 2, p. 7)

Other participants commented on having financial support from family and how this was a facilitator in the continuation of their medication regimen. One participant said:

…*it costs $60 in total. It is a bit pricey in my opinion, you know just to cough up $60 I think it’s every 30 days…I don’t think it would stop me so I am lucky at the moment that mum will help me if I need it to like, she’ll lend me some money to go buy it*...

(Participant 20, p. 5)

Some participants also noted having to purchase expensive medications at regular intervals to manage their condition. For instance, one participant who feels strongly obliged to bear the costs of their medication for the sake of their children’s health cited:

*Because there’s so many (medications) and constantly going to the get scripts and the money of that is huge because I don’t have a healthcare card…I hate it but it’s kind of like, I mean the cold sores (medication) is definitely worth it, nobody else in the family has them and I do not want to pass that onto my children*.

(Participant 23, p. 4)

Furthermore, participants appeared to justify the high price of medications if they perceived the medication to be effective in managing their health concern. It seems as though the notion of “value for money” was an underlying factor in participants’ motivations to adhere to an expensive medication. For example, one participant, who was seeking to change their medication, commented:

*I was on another one and I went to the doctor and said I’m not fussed about paying the extra money, I would like to go back on *(contraceptive pill type)* because I prefer that it’s shortened my periods*…

(Participant 24, p. 2)

#### 3.2.4. Sense of Agency

The theme “Sense of Agency” describes an underlying sense of importance or responsibility expressed by participants to have knowledge and understanding about their prescribed medication, side-effects, and impacts of non-adherence. Participants appeared to value being involved in these processes and having a sense of control. Most participants seemed to have some degree of knowledge about their medication and its impact on them. For example, one participant noted:

*I take them with a glass of water because if I don’t it does make my stomach quite sore, makes me feel quite ill … so I do have to have a lot of liquid and I always eat with them*…

(Participant 10, p. 4)

Participants with more complex regimens, however, appeared to have a deeper level of knowledge. For instance, one participant with a complex regimen commented:

…*it works for twelve hours and it’s a stimulant, so obviously I take *(sleep medication)* because I can’t sleep so I’ve got to be really careful when I take the (stimulant-type medication) in the mornings. I don’t take it past like nine AM.*

(Participant 22, p. 4)

Some participants expressed having undertaken their own research to gain more knowledge about their medication. One participant, who appeared to be unwilling to accept their doctor’s recommendation and preferred to do their own research said:

…*I get really bad headaches. And one doctor basically said that ‘Oh take this medication’. He didn’t really tell me what it was or what it was for. But I went home, had a Google, read up, and it was like basically epilepsy medication … that’s a bit of a big medication just to throw out there and tell me to take. Because that’s going to alter a lot of things. So yeah I take doctor’s advice with a high grain of salt*.

(Participant 6, p. 7)

##### Choice

In this subtheme of “Sense of Agency”, it seemed as though most participants valued having a choice in the medication they were taking and the reasons for taking that medication. A sense of ownership around medication choices may be tied to participants’ adherence to their medication. For example, one participant noted how they made their own executive decisions:

*I know a while back he kind of started thinking about taking me off it *(medication)*. But then like I said, sometimes I space out a bit so I thought I’d prefer to just stick to it for like the foreseeable future anyway*.

(Participant 3, p. 6)

#### 3.2.5. Adverse Outcomes

The theme “Adverse Outcomes” relates to participants experiences of negative bodily consequences, either related to the symptoms of their health condition or as side-effects of their medication regimen. These outcomes seemed to be experienced by all participants to some degree.

##### Symptoms of the Health Condition

In this subtheme, many participants described their experiences with the symptoms of their health condition, and how these symptoms functionally impacted areas of their life. For instance, one participant who takes medication for asthma said

…*I kind of get worried that I’m going to get sick or something and if that gets onto my chest, I won’t perform as well in this test or in this *(sporting)* competition*…

(Participant 20, p. 8)

Similarly, many participants spoke of their intention to adhere to their medication regimens to ensure they reduce their chances of experiencing these negative symptoms. For example, one participant stated:

…*if I miss a day or two like that, the pain *(period pain)* doesn’t come straight away which is good. But I do get like bleeding and stuff which is irritating and makes me want to be more regular with it.”*

(Participant 2, p. 8)

##### Side-Effects

This subtheme encompasses the participants’ experience of side-effects related to taking their medication or the side-effects and consequences experienced if the medication dosage is missed. For instance, one participant commented on their negative experience:

…*the main side effect of *(medication)* is dryness everything and I’ve already got quite a few issues with blood noses…when I get sick I can burst blood vessels quite easily in my nose and those will, I’ll have quite a few blood noses…so I got sick and I just kept getting little blood noses and I thought what was it and then I realised it was *(medication)* that was causing it *(the sore)* to not heal properly.*

(Participant 24, p. 4)

Furthermore, participants who experienced side-effects from taking their medication discussed that they continued to take the medication, despite the side-effects, for example:

*I would wake up in the mornings and just feel like I couldn’t do anything and that I needed to sleep. But I pushed through that*

(Participant 16, p. 1)

Some participants also chose to no longer adhere as a direct result of their negative experience. For example, a participant expressed:

…*it’s *(the sore)* like a small cut in my nose that’s not going away. That’s why I’m not taking it.*

(Participant 24, p. 5)

Contrary to not adhering to medication to avoid side-effects, some participants discussed how the negative consequences of missing the medication motivated them to adhere. Specifically, one participant expressed:

…*I mean with the pill you might get your period and like spotting and you might get pregnant which is also scary…I’ve always been very, very, very careful *(taking the medication)*…it’s if you forget one or two you’re not protected, end of story*.

(Participant 5, p. 2)

#### 3.2.6. Weighing Up Pros and Cons

The theme “Weighing Up Pros and Cons” encompasses participants’ process of considering what the outcomes of taking medication would be for them. This theme seems to underlie the five previously identified themes. This process appeared to be salient for all participants, regardless of regimen complexity. For example, one participant spoke of their experience of initial negative medication side-effects:

…*okay I might not actually be about to die. So I’ll keep going and then it *(muscle spasm side-effect of medication)* went away after about three weeks. And then after that I was like ‘yeah super worth that initial rough period*.

(Participant 1, p. 2)

Although this participant noted some initial undesirable side-effects, it appeared they perceived more value in the positive outcomes of the medication, and therefore intended to continue taking it. Some participants identified more negative factors, such as personal conflicts, when deciding whether to take medication. For instance, one participant who appeared to hold attitudes of pharmaceuticals as being harmful said:

…*the whole nausea aspect of it, and I just don’t know like taking something that I could not take, like putting chemicals into my body that I don’t necessarily need to. Like I don’t love that, but I think the benefits outweigh the negatives.”*

(Participant 4, p. 4)

Alternatively, other participants spoke about having considered both the positive and negative aspects of continuing their medication but suggested the benefits of the medication were a focal factor in their decision making. For example:

*It tastes disgusting, that’s probably the only thing I hate but that would never deter me from taking it because that’s how happy I am with taking it and the results that it provides me with.*

(Participant 11, p. 10)

Some participants also cited their explicit need for the medication to manage their condition. Thus, part of a participant’s process of justifying their medication use may be a reliance on their medication for quality everyday functioning. For example, one participant said:

…*without it I probably wouldn’t be able to function too well. I’d be able to function but my mood would be up and down. You know quite unhappy. Easy to rile up you know, get angry, and just the way I would respond and things like that. So, for me, it was I knew I had to do it*…

(Participant 8, p. 4)

See Table 1 for a brief description of the themes and subthemes.

## 4. Discussion

The findings partially supported temporal self-regulation theory, such that the importance of some variables (intention, cues, past behaviour, planning) were identified as being important and thus may facilitate adherence. Differences in simple and more complex regimens seemed particularly salient in the role of routines, planning and sense of agency, such that those with more complex regimens had more set routines, expressed higher importance and engagement in planning to ensure organisation and appeared to express greater knowledge related to their regimen.

### 4.1. Temporal Self-Regulation Theory

It appeared as though there was an underlying sense of motivation to want to adhere to medication regimens, even though participants seldom explicitly stated this intention. This is consistent with the theory, as temporal self-regulation theory suggests intention is important in executing a behaviour [18]. Furthermore, facets of both behavioural prepotency and self-regulatory capacity were identified. These factors appeared to manifest where participants discussed the role of routines and frequent engagement in the behaviour (which is similar to past behaviour), and cues in assisting them to adhere, as shown in the “Routines” theme and related subthemes. Health professionals should consider advising patients starting new medications of the importance of a consistent routine, such that medications should be taken at the same time each day to ensure consistent and repeated execution of the behaviour such that it may become automatic over time [21].

Another facet of the theory that was commonly expressed was the role of cues. The benefits of visual cues have been previously quantitatively identified [34], however, the role of different types of cues in improving adherence has only recently been explored in relation to improving adherence [28]. Our study further supports the notion that visual, contextual, and sensory cues may encourage adherence and aid in reducing forgetfulness, especially for those using only one medication. Concerning self-regulatory capacity, planning was the most saliently discussed facet and appeared as a subtheme in “Routines”, however, there were also underlying notions of self-control in the “Sense of Agency” and “Routines” themes. These findings are not unusual, as previous research has shown planning [35], along with the perceived ease and feeling confident in enacting the behaviour (i.e., self-efficacy), can facilitate medication adherence [36].

Interestingly, and in contradiction to the theory, few participants mentioned adherence as being habitual, however, many discussed the importance of routines. This is possibly due to habit being defined as an unconscious process whereby participants have difficulty identifying their behaviour as a habit [37]. However, often habits and routines are referred to interchangeably in the psychology domain as they both refer to regular and repeated actions [38,39], and so this may just be a matter of semantics.

### 4.2. Simple vs. More Complex Regimens

The importance of having a consistent routine appeared to be more salient for participants with more complex regimens, rather than those with simpler regimens. This notion is consistent with previous literature that suggests having a daily routine is important when taking multiple medications, to promote more effective adherence [40]. Similarly, planning was more commonly mentioned by participants with more complex regimens. This is a novel finding and lends itself to being further investigated to see whether planning significantly facilitates adherence in medication regimens. To assist planning for those with complex regimens, the use of mobile prescription management apps, such as MedAdvisor [41], may ensure that prescriptions do not run out and are refilled in time. Health professionals and pharmacists should both consider promoting this option, which is said to increase adherence by approximately 20% [41].

The use of cues seemed to vary across participants of different regimen complexities. Those with simple regimens appeared to rely more on the use of visual cues (e.g., medication box on bed), whilst it appeared those with more complex regimens relied on contextual cues, such that taking one medication was suggested by participants to prompt taking other medications. Future research may consider further exploring the role and effectiveness of cues by conducting experimental studies that ask participants engaged in simple medication regimens (such as the oral contraceptive pill) to choose a specific cue to pair with taking their medication. However, in the meantime and in light of these findings and previous findings by Orr and colleagues [34], health professionals should consider promoting the use of visual or contextual cues with taking medications to their patients to assist in possibly ensuring greater adherence.

In addition, although not a facet of temporal self-regulation theory, there appeared to be differences in knowledge and knowledge-seeking behaviour between those with simple and more complex regimens. Those with more complex regimens appeared to express a greater degree of knowledge and knowledge-seeking related to their condition and specific medications. Disease and medication specific knowledge is said to be strongly and positively associated with health literacy [42], which may suggest that our sample is not highly knowledgeable in their specific disease or medication, but rather are more health literate in general. However, the relationship between health literacy and medication adherence has not been explored extensively [43], even in simple regimens, and may provide avenues for future research in this area.

### 4.3. Non Temporal Self-Regulation Theory Variables

The findings also identified several variables influencing adherence that are not included in temporal self-regulation theory. The cost of medications was not a barrier to adherence, which is inconsistent with the large majority of previous research in this area [44]. This may be due to the Australian Pharmaceutical Benefits Scheme (PBS) [45] which subsidises the cost of medications for most health conditions, making them more affordable and therefore accessible. Thus, research related to the cost of medications in the US and other countries may not be applicable in an Australian context.

Furthermore, the avoidance of negative symptoms of the health condition seemed to be associated with adherence. This disease-specific factor is slightly different to what has been identified in previous research, which tends to suggest that individuals view medication adherence as being necessary to be able to cope with their condition [33,46]. The role of avoidance of negative symptoms in facilitating medication adherence is common for those taking prophylactic medication (e.g., to prevent asthma symptoms) [47], however, it should be further studied in those taking medications to treat a condition, such as antidepressants. Perhaps incorporating questions explicitly asking individuals if they take their medication to avoid possible negative symptoms of their condition, is one way to explore this further. The importance of having support networks, both professional and social, was also identified, which is not unusual [48]. However, increased support has shown to be conducive to better adherence [33] which was not explicitly stated in our study. Many participants suggested they did not care too much about the lack of support or stigma they received and continued with their regimen regardless.

The use of an inductive and deductive approach has allowed for the identification of these important variables that are not part of temporal self-regulation theory. This provides opportunities for future research to test temporal self-regulation theory quantitatively, but with the addition of these variables to explore whether they can predict additional variance in medication adherence beyond that of the theory. Such extensions to theories are common in the literature [15].

### 4.4. Limitations

While the current study was successful in identifying different psychological variables as being important in different regimen complexities, the study is not without its limitations. The most salient limitation is that we were unable to capture a sample with “complex” regimens, with only five participants having between three and five medications. It appears that our study captured a sample of “simple” (one or two medications) and “not so simple” medication regimens. Related to this, our sample was a university sample and these samples tend to be younger and more highly educated compared to the general public [49]. This may explain why there was a low number of participants who expressed non-adherence because they “know better” and perhaps have greater health literacy. It may also explain why knowledge and knowledge-seeking was a salient theme throughout the findings. However, although we captured fewer complex regimens than we would have liked, we still had a degree of complexity with participants taking numerous medications. This study has been able to shed light on an under researched, yet important, area of adherence. The findings show there are differences in what is viewed as being important in adhering to simple and more complex regimens, despite the limited complexity of our sample. Future research should take into account these important preliminary findings, and apply temporal self-regulation theory quantitatively and qualitatively to samples engaged in operationally defined simple regimens and complex regimens to explore whether these differences are also apparent.

## 5. Conclusions

The present study sought to explore the utility of temporal self-regulation theory in helping to explain medication adherence in people’s daily lives, specifically in how they adhere to their medication regimens, and whether there are any differences in how the theory operated in adherence to different medication regimen complexities. Six themes that influence adherence were identified. Differences between regimen complexities appeared, such that participants who take between three and five medications spoke more on the importance of having a consistent routine, planning and seeking knowledge. Participants taking only one medication highlighted the importance of implementing cues, specifically visual, to assist in adherence. The findings show some support for temporal self-regulation theory, specifically intention, past behaviour, cues and planning, but many non-psychological influences were also identified, such as the cheap cost of medications, support from health professionals and friends, the experience of side-effects, avoiding negative symptoms of the condition and being involved in the process. However, complex regimens were not necessarily captured in the university sample and therefore future research should consider applying the theory to samples with distinct simple and complex regimens. Future research may also consider investigating the role of visual or contextual cues in simple regimens to see if adherence can be improved over time.

## Figures and Tables

**Table 1:** Description of themes and related subthemes.

Theme	Subthemes	Description
Routines		Maintaining medication regimens in a consistent and routinised way
	• Planning	Putting plans in place to ensure preparedness
	• Cues	Use of prompts and reminders to ensure adherence
	• Lifestyle Factors	Lifestyle barriers and facilitators of adherence
External Supports		The influence of different support systems in participants’ adherence
	• Professional Support	Support from trusted health professionals
	• Social Support	Support from loved ones
	• Stigma	Prejudice and judgement from those around them
Cost		The different role of medication cost in adhering to regimen
Sense of Agency		Having a responsibility to understand and be knowledgeable about regimen
	• Choice	Making choices related to own medication and regimen
Adverse Outcomes		Experiences of negative bodily outcomes related to taking medication or lack thereof
	• Symptoms of the health condition	The influence of symptoms on adherence
	• Side-effects	Negative side effects from taking medication or missing medication
Weight up Pros and Cons		The process of considering the positives and negatives of the medication regimen

## Supplementary Materials

The following are available online at https://www.mdpi.com/2227-9032/8/4/487/s1, “Interview schedule—Medication adherence”.
