# Supplementary material for: A Qualitative Application of Temporal Self-Regulation Theory to Understand Adherence to Simple and Complex Medication Regimens
Source: Healthcare (Basel). 2020 Nov 16;8(4):487. doi: 10.3390/healthcare8040487 (PMC7711536; doi:10.3390/healthcare8040487)
Supplement: Supplementary file 1 [file healthcare-08-00487-s001.pdf]

healthcare MDPI

Supplementary Material

Interview schedule – Medication adherence

- Interviewer obtains consent to audio-record the interview.
- Interviewer also re-iterates that the study is voluntary; the participant does not need to answer a question if he/she does not want to, and that he/she can also withdraw at any time without penalty.
- Rapport will be built prior to asking participant first question.
- Participant will have to complete consent and demographics on Qualtrics prior to interview

1. Can you begin by telling me about your current medication usage and needs?
    a. Can you tell me what each of these medications is for and how they are taken?
    b. Do you have a set routine for taking these?
    c. Do you find them easy or hard to take?

2. How do you feel about taking these medications?
    a. Can you tell me why/why not you think taking medication is important?
    b. How do your friends and family feel about you taking these medications?
    c. Can you tell me about your motivations for adhering or not-adhering to your medication?
    d. What do you think would happen if you didn't take your medication?
    e. Is there a medication you take that you think is more important than the others?
        a. Why?
3. How would you describe your overall adherence to these medications?
    a. Do you take them as you have been directed by your health professional?
    b. Sometimes people simply forget to take their medication, do you sometimes experience this?
    c. People also deliberately sometimes do not take their medication, do you sometimes do this?
        i. If so, why?
    d. What are some things that make it easier for you to take your medication?
        i. Are there any particular things or techniques you have in place to assist you?
    e. Is there anything that makes it a bit harder? Such as things that get can get in the way of you taking your medication correctly
        i. Can you please tell me about them – how influential are they?
        ii. Is there anything that could be improved that would change this? E.g. cost of medication
        iii. What do you think would assist you in improving your adherence?

4. What is your experience of side-effects from medication?
    a. Please elaborate – type, number, when
    b. Did this influence you views/motivations for taking medication?

c. Did it influence your adherence? How?

© 2020 by the authors. Submitted for possible open access publication under the terms and conditions of the Creative Commons Attribution (CC BY) license (http://creativecommons.org/licenses/by/4.0/).
